# Tumor Characteristic Variations between Symptomatic and Asymptomatic Endometrial Cancer

**DOI:** 10.3390/healthcare9070902

**Published:** 2021-07-16

**Authors:** Petra Vinklerová, Markéta Bednaříková, Luboš Minář, Michal Felsinger, Jitka Hausnerová, Petra Ovesná, Vít Weinberger

**Affiliations:** 1 Department of Gynecology and Obstetrics, University Hospital Brno, Masaryk University, 60177 Brno, Czech Republic; Vinklerova.Petra@fnbrno.cz (P.V.); Minar.Lubos@fnbrno.cz (L.M.); Felsinger.Michal@fnbrno.cz (M.F.); 2 Department of Internal Medicine, Hematology and Oncology, University Hospital Brno, Masaryk University, 60177 Brno, Czech Republic; Bednarikova.Marketa@fnbrno.cz; 3 Department of Pathology, University Hospital Brno, Masaryk University, 60177 Brno, Czech Republic; Hausnerova.Jitka@fnbrno.cz; 4 Faculty of Medicine, Institute of Biostatistics and Analyses, Masaryk University, 60177 Brno, Czech Republic; ovesna@iba.muni.cz

**Keywords:** endometrial cancer, tumor markers, ER, PR, p53, L1CAM

## Abstract

Endometrial cancer is the most common gynecologic malignancy in Europe and usually diagnosed in its initial stage owing to early symptoms of abnormal bleeding. There is no population screening for this disease, although it can sometimes be accidentally diagnosed in asymptomatic patients. Our study aims to determine differences in clinical and tumor characteristics between an asymptomatic and symptomatic group of patients. This unicentric prospective observational study took place in University Hospital Brno between January 2016 and December 2019. A total of 264 patients met inclusion criteria (26% asymptomatic, 74% with reported symptoms). We did not find a statistically significant difference in clinical characteristics (menopausal status, parity, age, BMI, and serum level of CA 125) between groups. According to ultrasound examination, bleeding tumors were larger (19.5 vs. 12.7 mm, *p* ≤ 0.001). Definitive histology results indicated more frequent lymphovascular space invasion (*p* < 0.001), along with deep myometrial (*p* = 0.001) and cervical (*p* = 0.002) invasion. There was no difference in advanced stages of the tumor. We did not substantiate statistically significant difference in immunohistochemical profile (estrogen and progesterone receptors, L1 cell adhesion molecule, tumor protein p53), which is relevant for tumor recurrence risk and survival capacity. Our conclusions affirmed that bleeding occurs more often among patients with local tumor invasion into the myometrium and cervical stroma. Final International Federation of Gynecology and Obstetrics (FIGO) stage, histology, and immunohistochemical characteristics do not significantly affect symptom appearance.

## 1. Introduction

Endometrial cancer (EC) is the most common gynecologic malignancy in Europe, and incidence is increasing [1]. It is usually diagnosed in the first or second stage in accordance with The International Federation of Gynecology and Obstetrics (FIGO) 2009 classification. Generally, prognosis is favorable owing to initial symptom visibility; abnormal bleeding or spotting. The cancer can be unintentionally recognized among symptom-free women with thickened endometrium on an ultrasound scan or in a uterus specimen following a hysterectomy executed for other reasons (uterine prolapse, uterine fibroids, etc.). Population screening for this cancer does not exist, and routine biopsy of the asymptomatic patient with endometrial polyps or thickened endometrium is not recommended [2,3]. Nevertheless, in many countries where ultrasound is routinely used in daily practice, ordering a biopsy with asymptomatic patients is common after recognizing specific abnormal ultrasound images. Even women with no signs of bleeding can therefore be diagnosed with incidental endometrial cancer.

Currently, it is not only the disease expansion (FIGO stage), the histologic type, lymphovascular space invasion (LVSI), and grade that determines the tumor’s biological nature. The recently introduced endometrial cancer classification is based on molecular characteristics defined by The Cancer Genome Atlas (TCGA) research group [4]. According to TCGA, endometrial tumors are divided into four groups consistent with their mutation profile. This correlates with prognosis and expected response to the adjuvant treatment: POLE (polymerase-epsilon) ultramutated, hypermutated (microsatellite instable, MSI), copy-number low (microsatellite stable, MSS), and copy-number high (serous-like). Broad implementation of a method into common practice is hindered by availability and cost, even in western countries. A combination of a molecular profile (POLE exonuclease domain mutation analysis) with an immunohistochemical one (p53, MSH6, and PMS2) appears to be a means of comprehensively incorporating a new classification [5,6].

Besides molecular classification (TCGA), a variety of prognostic immunohistochemical markers exist with a proven impact on disease-free survival and overall survival in endometrial cancer patients. The L1 cell adhesion molecule (L1CAM) is recognized as an important predictor and risk factor for recurrence and poor survival with both early-stage and advanced endometrial cancer [7,8]. Moreover, the mutational status of tumor protein p53 is associated with an unfavorable outcome [5]. Conversely, high levels of estrogen receptors (ER) and progesterone receptors (PR) expression are associated with early-stage tumors, and excellent overall disease-specific survival [9,10]. Loss of these receptors, however, is a significant predictor of lymph node metastasis and poor prognosis [11,12].

Our study intent was to identify potential differences between symptomatic and asymptomatic tumor characteristics. What kind of endometrial malignancy is evidenced with symptoms of bleeding, spotting and which carcinoma could be asymptomatic? Is symptomatology a strong marker of any specific kind of endometrial cancer? Our research recorded clinical and histological tumor characteristics in patients with and without symptoms (abnormal bleeding/spotting). While focusing on traditional characteristics such as histological type, grade, and stage of the tumor, we also included information on immunohistochemical markers, which were previously associated with poor prognosis (L1CAM positivity, mutation of p53, loss of hormone receptors ER, PR).

## 2. Materials and Methods

Our prospective observational study took place between January 2016 and December 2019 at the Department of Gynecology and Obstetrics, University Hospital Brno, Czech Republic. With local hospital ethics committee approval, all patients signed an informed consent. Patients who underwent surgery treatment following their endometrial cancer diagnosis were included in the study. Patients were placed into either a symptomatic or asymptomatic group depending on definitive initial symptoms including postmenopausal bleeding, spotting, pinkish discharge, or irregular and excessive bleeding in premenopausal women.

Women were termed postmenopausal when their last menstruation period was at least one year prior to the diagnosis. Exclusion criteria were no surgical treatment, uterine sarcoma histology, unknown symptomatology status.

All patients diagnosed with endometrial cancer after preoperative biopsy underwent an expert transvaginal and transabdominal ultrasound examination as a part of the mandatory staging procedures before surgery. International Endometrial Tumor Analysis (IETA) terminology was used to define uterine pathology [13]. Tumor size was detailed in three dimensions, and the largest one was used for analysis.

All patients endured total abdominal or laparoscopic hysterectomies with bilateral salpingo-oophorectomies (ovaries spared when patients were younger than 45 years). Pelvic and paraaortic lymphadenectomies were effected consistently with recent recommendations [2] and patient performance status. An infracolic omentectomy was employed when cases embodied serous carcinoma or carcinosarcoma.

Clinical patient characteristics (age, menopausal status, body mass index, parity, preoperative serum level of CA 125) were recorded and obtained from medical files. 

Histological type (endometrioid, mucinous, serous, clear-cell, carcinosarcoma), grade, lymphovascular space invasion (LVSI), myometrial and cervical invasion, immunohistochemical profile (ER, PR, L1CAM, p53), and pathological stage (according to FIGO 2009) were obtained from pathology reports. Within the immunohistochemical profile, we used standard cut-offs for positivity of ER, PR, L1CAM (>10%) [11,14] along with cut-offs we found more accurate in our previous study (≥88%, ≥78%, and ≥4%) [12].

Categorical variables were summarized as absolute and relative frequencies; differences between two or more groups were compared using the maximal likelihood chi-square test. Bonferroni correction was applied to post-hoc testing. Continuous variables were listed as mean, or geometric mean in the case of log-normally distributed data, accompanied by a 95% confidence interval (CI). Comparison between groups was recorded using a t-test. All tests were two-sided at a 0.05 level of significance. Analysis used IBM SPSS Statistics.

## 3. Results

Between 2016 and 2019, 313 patients were diagnosed with uterine cancer at the Department of Obstetrics and Gynecology, University Hospital Brno, Czech Republic. Two hundred and sixty-four of these patients met inclusion criteria. Sixty-nine women (26%) were asymptomatic, and 195 (74%) reported symptoms. We did not find a statistically significant difference in clinical characteristics (menopausal status, parity, age, BMI, and CA 125 serum level) between the symptom-free and symptomatic groups (Table 1).

**Figure 1:**
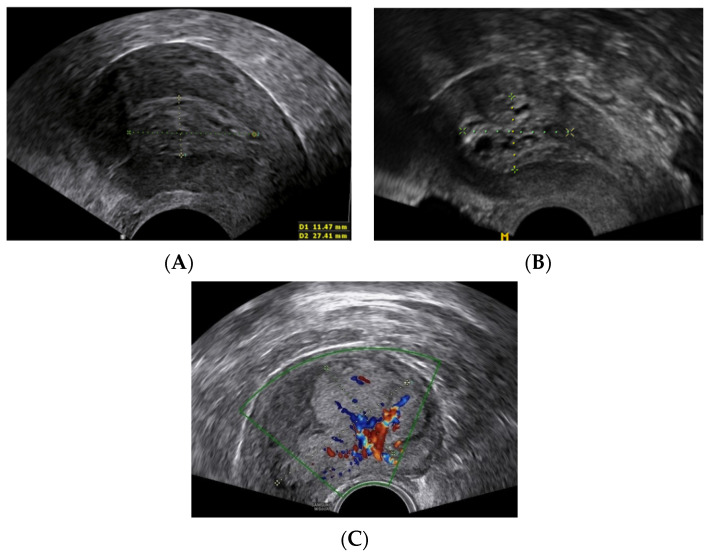
Tumor ultrasound characteristics. (**A**) No myometrial invasion; (**B**) myometrial invasion <½; (**C**) myometrial invasion >½.

All 264 patients received preoperative ultrasound scans which indicated that symptomatic tumors were significantly larger than asymptomatic (19.5 vs. 12.7 mm, *p* ≤ 0.001, Table 2, Figure 1).

From the perspective of tumor type and differentiation, asymptomatic ones were more common endometrioid grade 1 (*p* ≤ 0.001). Poorly differentiated and non-endometrioid tumors were more frequently bleeding (endometrioid grade 3, *p* = 0.001 with non-endometrioid tending towards a statistical significance of *p* = 0.090, Table 3). Definitive histology results clearly confirmed that deep myometrial (*p* = 0.001) and cervical (*p* = 0.002) invasion was much more common in the symptomatic group, which corresponds with a higher incidence of stage IB and II in those patients. LVSI of the tumor was more frequently recognized in the symptomatic group (*p* < 0.001). On the other hand, our final histology indicated that asymptomatic tumors were more likely to be at a FIGO 1A stage (*p* ≤ 0.001), whereas symptomatic tumors were those which ultimately invaded cervical stroma (FIGO II, *p* = 0.017). With FIGO IB (*p* = 0.549) and other stages, there was no statistically significant difference between the two groups (Table 3).

**Figure 2:**
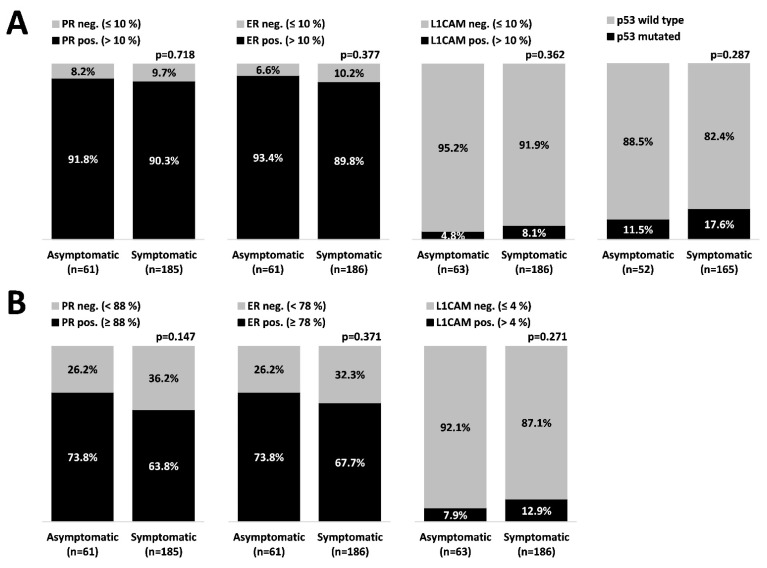
Tumor immunohistochemical profile. (**A**) A standard cut-off [11,14]; (**B**) an alternative cut-off [12].

Overall, 249 tumors with desired immunohistochemical characteristics were detected; 63 asymptomatic and 186 symptomatic. Based on cut-off data (Figure 2A), we did not determine any statistically significant difference in immunohistochemical profile (ER, PR, L1CAM, p53) between groups (even though we used also alternative cut-offs, as seen in Figure 2B).

## 4. Discussion

A majority of endometrial cancers (80%) are diagnosed in the early stage (FIGO stage I) with five-year overall survival rates of over 95% [15]. The principal risk factors for endometrial cancer are age, nulliparity, and obesity. Most tumors (90%) are found in postmenopausal women with a mean age at diagnosis of 62 years [16]. Only 4% of endometrial carcinomas are diagnosed in women younger than 40 years [17]. Endometrial cancer risk increases 2.6-fold with a BMI > 30 and increases 4.7-fold with a BMI > 35 compared to women with moderate weight (BMI < 25) [18]. Parity reduces EC risk (RR 0.66, Cl 0.6–0.74) [19].

Serum marker CA 125 is preoperatively elevated in low-risk EC with a higher risk of recurrence, in high-risk carcinomas (endometrioid grade 3, non-endometrioid), and in lymphatic node involvement cases [20].

In our cohort, we did not determine a difference between the symptomatic and symptom-free group of EC in terms of menopausal status (*p* = 0.71), nor did we recognize a difference between the groups from the point of view of parity (*p* = 0.675), age (*p* = 0.846), BMI (*p* = 0.451), or preoperative CA 125 serum level (*p* = 0.649).

Bleeding or spotting occurs in up to 90% of women with endometrial cancer [21]. However, this symptom is common and potentially triggered by many other pathologies, so only 9% of women with postmenopausal bleeding are diagnosed with endometrial cancer [21]. Among premenopausal women with abnormal or irregular bleeding, the risk is much lower (0.33% for EC; 1.31% for EC or atypical hyperplasia) [22].

Ultrasound is the preferred imaging method among gynecologists, used during routine annual examinations and with symptomatic gynecological pathology cases. Anamnestically, the issues of abnormal premenopausal hemorrhage and any postmenopausal spotting or bleeding are very important. In these cases, the most common pathology detected by ultrasound is endometrial hyperplasia or uterine polyp. Based on symptoms and ultrasound scan results, a biopsy may be indicated.

The easiest means of histological verification among outpatients is to use a pippel. In elderly women with vaginal atrophy or cervical stenosis, this procedure can be challenging, and biopsy by dilatation and curettage (D&C) under general anesthesia is necessary. A hysteroscopy is recommended to take a representative sample and remove the focal lesion, however complication potential poses risks (uterine perforation, bowel damage, bleeding, infection, fluid-overload syndrome, etc.) [23,24]. With this perspective, risks and benefits of invasive diagnostic procedures are evaluated for each particular case as we assess objective ultrasound results in relation to specific patient symptoms.

There is a 12% prevalence of thickened endometrium ≥5 mm in gynecologically healthy asymptomatic postmenopausal women [24]. The risk of malignancy is only 0.4% in these patients and increases in accordance with endometrial thickness [25]. Our study has recognized a statistically significant difference in tumor size in the EC asymptomatic group compared to the symptomatic group (*p* ≤ 0.001). Assessing our results in detail, the average size of the asymptomatic tumor was clearly 12.7 mm, which is consistent with other study references, where a threshold of ≥11 mm is deemed suitable for biopsy in symptom-free patients [25,26]. EC risk with this endometrial thickness is 6.7% [25]. Still, 19 unnecessary endometrial samplings were undertaken to detect one endometrial cancer or atypical hyperplasia in this patient group [26].

Endometrial polyps are found in 10–40% of reproductive-age women examined by ultrasound [27]. With abnormal bleeding or infertility, a hysteroscopy is recommended. On the other hand, among asymptomatic patients, conservative management is more appropriate, since malignancy risk is low and small polyps can regress spontaneously [28]. According to a large meta-analysis, EC risk is higher in patients with bleeding (5.14%) compared to the asymptomatic (1.89%), and EC risk is greater with postmenopausal (4.93%) women compared to premenopausal women (1.12%) [29].

Our study also focused on identifying histopathological and immunohistochemical differences in characteristics of symptomatic and asymptomatic uterine malignant tumors. Asymptomatic carcinomas were more frequently endometrioid grade 1 (49.3% vs. 20.5%, *p* < 0.001), while bleeding tumors were categorized as endometrioid grade 3 (14.9% vs. 0%, *p* = 0.001). No significant differences prevailed between these two groups with other histological types.

According to our results, it is evident that an asymptomatic condition facilitates local tumor growth and eventual cervical and, most notably, myometrium penetration. Even advanced stages were ascertained to be asymptomatic without extensive local spread into the cervix and myometrium. While symptomatology is linked to local uterine invasion, its relevance to lymphatic node metastasis or distant involvement (FIGO III, IV) has not been evidenced. In the study of Gemer et al., the authors assembled a large multicentric retrospective cohort, where there were proportionally more patients diagnosed at stage IA (compared with IB) in the asymptomatic group (82% vs. 66% in symptomatic; *p* < 0.001) [30]. Thus, instead of histology and grade, bleeding and spotting symptoms seemed to be contingent upon local tumor invasion characterized by lymphovascular invasion (*p* ≤ 0.001), size (*p* ≤ 0.001), penetration into the myometrium (*p* = 0.001) and the cervix (*p* = 0.002).

Loss of hormonal receptors ER and PR is associated with a high risk of lymph node metastases, reduced DFS (disease-free survival), and DSS (disease-specific survival) [11,31]. The expression of L1CAM is associated with advanced stage, nodal involvement, high grade, non-endometrioid histology, and LVSI. Moreover, there is a high risk of distant recurrence and reduced survival in endometrioid EC [14]. P53 immunohistochemistry precisely reflects the tumor’s TP53 mutation status. This is the single most important molecular factor predicting prognosis in endometrial carcinomas, with the presence of a TP53 mutation being associated with an unfavorable outcome [5,32].

Unfortunately, we did not substantiate statistical difference between our two groups (symptoms versus symptoms-free) with regards to the ER, PR, and L1CAM status. Recognizing that hormonal receptors’ cut-offs (10%) are based on breast cancer data and, therefore, a potential source of bias, we sought to apply newer, more accurate cut-offs previously published by our group [11] pursuant to the recently published European Network for Individualized Treatment of Endometrial Cancer (ENITEC) collaboration study. The authors divided endometrial cancer into three prognostic groups based on ER/PR expression: high-risk (0–10%), intermediate-risk (20–80%) and low-risk (90–100%) [33]. Even after employing alternative cut-offs for ER, PR, and L1CAM of 88%, 78%, and 4% respectively, we did not record any significant difference between symptomatic and asymptomatic groups. Identical intriguing non-statistically significant results were reported with respect to p53 mutation status.

We postulate that bleeding and spotting in patients with uterine carcinoma primarily depend on local tumor progression. Final FIGO stage, histology, and immunohistochemical characteristics do not play a key role. This statement, however, needs to be substantiated with a larger study cohort. Moreover, it would be interesting to compare and distinguish patient symptoms with reference to TCGA tumor characteristic classification, which is currently the most accurate grouping system, albeit without relevant symptom differentiation data at this point in time.

As far as we know, our study is the first to identify endometrial tumor characteristics while simultaneously including an immunohistochemical profile of symptomatic versus asymptomatic patients. Our timely reporting describes complex clinical characteristics, preoperative ultrasound scan results, and histopathological records including IHC. In the future, we look towards improving unicentric design and including more data from advanced uterine carcinomas FIGO III and IV, while more precisely detailing symptom durations. This could be very important and relevant information in relation to local tumor progression, even though some studies did not find a difference, for example between asymptomatic patients compared to those with symptoms less than 3 months and more than 3 months [34].

## 5. Conclusions

Our study has determined that symptomatology occurs more often among patients with invasive local tumors impacting their myometrium and cervical stroma. Final FIGO stage, histology, and immunohistochemical characteristics do not play a key role in symptom appearance among patients with endometrial cancer. Bleeding and spotting are neither a strong marker nor a sign of any specific kind of endometrial carcinoma.

## Figures and Tables

**Table 1:** Patients’ clinical characteristics.

	Asymptomatic (*n* = 69)	Symptomatic (*n* = 195)	*p*-Value
	n (%)	n (%)	
Menopausal status			0.071
Premenopausal	5 (14.3%)	30 (85.7%)	
Postmenopausal	64 (27.9%)	165 (72.1%)	
	*mean (SD)*	*mean (SD)*	
Parity (*n* = 69/195)	1.8 (0.9)	1.8 (0.9)	0.675
Age (*n* = 69/195)	65.2 (9.22)	64.9 (11.21)	0.846
BMI (*n* = 67/195)	31.6 (6.58)	32.4 (7.08)	0.451
CA 125 ^1^ (*n* = 45/155)	17.1 (1.83–160.87)	18.4 (3.47–97.73)	0.649

^1^ Geometric mean (95% confidence interval).

**Table 2:** Tumor ultrasound characteristics.

	Asymptomatic (*n* = 69)	Symptomatic (*n* = 195)	*p*-Value
	*mean (SD)*	*mean (SD)*	
The largest tumor dimension (mm) ^1^	12.7 (3.56–45.36)	19.5 (4.88–78.31)	<0.001

^1^ Geometric mean (95% confidence interval).

**Table 3:** Tumor histological characteristics.

	Asymptomatic (*n* = 69)	Symptomatic (*n* = 195)	*p*-Value
	*n* (%)	*n* (%)	
Histological type and grade ^1^			<0.001
Endometrioid grade 1	34 (49.3%)	40 (20.5%)	<0.001
Endometrioid grade 2	31 (44.9%)	100 (51.3%)	0.364
Endometrioid grade 3	0 (0%)	29 (14.9%)	0.001
Non-endometrioid	4 (5.8%)	26 (13.3%)	0.090
Lymphovascular space invasion			<0.001
No	66 (95.7%)	151 (77.4%)	
Yes	3 (4.3%)	41 (21%)	
Myometrial invasion			0.001
None or <½	62 (89.9%)	137 (70.3%)	
>½	7 (10.1%)	58 (29.7%)	
Cervical invasion			0.002
None	65 (94.2%)	154 (79%)	
Stromal invasion	4 (5.8%)	41 (21%)	
Pathological stage (FIGO 2009)			<0.001
IA	59 (85.5%)	118 (60.5%)	<0.001
IB	6 (8.7%)	22 (11.3%)	0.549
II	3 (4.3%)	30 (15.4%)	0.017
IIIA	0 (0%)	4 (2.1%)	0.231
IIIB	1 (1.4%)	2 (1%)	0.775
IIIC	0 (0%)	12 (6.2%)	0.035
IVA	0 (0%)	0 (0%)	-
IVB	0 (0%)	7 (3.6%)	0.111

^1^ Mucinous histology was included in the endometrioid group.
